# Intramuscular anabolic signaling and endocrine response following high volume and high intensity resistance exercise protocols in trained men

**DOI:** 10.14814/phy2.12466

**Published:** 2015-07-21

**Authors:** Adam M Gonzalez, Jay R Hoffman, Jeremy R Townsend, Adam R Jajtner, Carleigh H Boone, Kyle S Beyer, Kayla M Baker, Adam J Wells, Gerald T Mangine, Edward H Robinson, David D Church, Leonardo P Oliveira, Darryn S Willoughby, David H Fukuda, Jeffrey R Stout

**Affiliations:** 1 Institute of Exercise Physiology and Wellness, Sport and Exercise Science, University of Central FloridaOrlando, Florida, USA; 2 Department of Internal Medicine, College of Medicine, University of Central FloridaOrlando, Florida, USA; 3 Exercise and Biochemical Nutrition Laboratory, Baylor UniversityWaco, Texas, USA

**Keywords:** Hormone hypothesis, intensity, mTOR pathway, mTORC1, volume

## Abstract

Resistance exercise paradigms are often divided into high volume (HV) or high intensity (HI) protocols, however, it is unknown whether these protocols differentially stimulate mTORC1 signaling. The purpose of this study was to examine mTORC1 signaling in conjunction with circulating hormone concentrations following a typical HV and HI lower-body resistance exercise protocol. Ten resistance-trained men (24.7 ± 3.4 years; 90.1 ± 11.3 kg; 176.0 ± 4.9 cm) performed each resistance exercise protocol in a random, counterbalanced order. Blood samples were obtained at baseline (BL), immediately (IP), 30 min (30P), 1 h (1H), 2 h (2H), and 5 h (5H) postexercise. Fine needle muscle biopsies were completed at BL, 1H, and 5H. Electromyography of the vastus lateralis was also recorded during each protocol. HV and HI produced a similar magnitude of muscle activation across sets. Myoglobin and lactate dehydrogenase concentrations were significantly greater following HI compared to HV (*P* = 0.01–0.02), whereas the lactate response was significantly higher following HV compared to HI (*P* = 0.003). The growth hormone, cortisol, and insulin responses were significantly greater following HV compared to HI (*P* = 0.0001–0.04). No significant differences between protocols were observed for the IGF-1 or testosterone response. Intramuscular anabolic signaling analysis revealed a significantly greater (*P* = 0.03) phosphorylation of IGF-1 receptor at 1H following HV compared to HI. Phosphorylation status of all other signaling proteins including mTOR, p70S6k, and RPS6 were not significantly different between trials. Despite significant differences in markers of muscle damage and the endocrine response following HV and HI, both protocols appeared to elicit similar mTORC1 activation in resistance-trained men.

## Introduction

Resistance exercise paradigms are often divided into high volume (HV) or high intensity (HI) protocols. HV protocols typically involve greater volume (3–6 sets; 8–12 repetitions), moderate intensities [<85% 1 repetition maximum (1-RM)], and short rest intervals (30–90 sec), while HI protocols typically involve higher intensities (≥85% 1-RM), low volumes (2–6 sets; ≤6 repetitions), and longer rest intervals (3–5 min) (ACSM, 2009). Although changes in the acute program variables surrounding a resistance exercise prescription have been suggested to promote differing anabolic responses and hypertrophic adaptation in skeletal muscle (Kraemer and Ratamess 2005), the optimal parameters of a resistance training program for regulating muscle growth remain unclear (Adams and Bamman 2012). The stimulus from muscle contraction during resistance exercise of differing intensities results in varying biochemical responses regulating the rate of protein synthesis (Hornberger 2011). At the cellular level, skeletal muscle adaptation is the result of the cumulative effects from transient changes in gene expression following acute bouts of exercise (Coffey and Hawley 2007). Thus, maximizing the resistance exercise-induced anabolic response stimulates the greatest potential for hypertrophic adaptation with training.

Acute program variables, including exercise intensity, volume, and rest interval, influence the endocrine response following resistance exercise (Kraemer and Ratamess 2005). Specifically, HV resistance exercise has been suggested to produce significantly greater elevations in both anabolic and catabolic hormones compared to HI resistance exercise (Kraemer et al. 1990; Hakkinen and Pakarinen 1993; Smilios et al. 2003; Linnamo et al. 2005; Crewther et al. 2008; McCaulley et al. 2009; Uchida et al. 2009). Systemic elevations of circulating hormones increase the likelihood of interaction with receptors located within muscle tissue and have been suggested to contribute to muscle growth consequent to resistance training (Kraemer and Ratamess 2005). However, the mechanisms of exercise-mediated muscle hypertrophy have been suggested to be solely an intrinsic process, which is not influenced by transient changes in circulating hormones (West et al. 2009, 2010a,b; Mitchell et al. 2013). Although a high volume, moderate intensity training protocol (i.e., designed to elicit muscle hypertrophy) is thought to stimulate a greater endocrine response, physiological fluctuations in ostensibly anabolic hormones have not been shown to enhance muscle protein synthesis (West et al. 2009), intramuscular anabolic signaling (Spiering et al. 2008; West et al. 2009), or resistance training-induced muscle hypertrophy (Mitchell et al. 2013).

The mammalian/mechanistic target of rapamycin complex 1 (mTORC1) signaling pathway appears to be the primary regulator of muscle protein synthesis and growth (Hornberger et al. 2006; Drummond et al. 2009; Goodman 2014). Phosphorylation of signaling proteins within the mTORC1 pathway regulates translation initiation, the rate-limiting step in protein synthesis (Welle et al. 1999; Brian et al. 2012). Insulin and growth factors lead to the phosphorylation of protein kinase B (Akt), which activates mTORC1 signaling by inactivating tumor sclerosis complex 2 (TSC2), a primary inhibitor of mTORC1 (Inoki et al. 2002). Resistance exercise also evokes a robust activation of mTORC1 signaling in human skeletal muscle (Coffey et al. 2006; Koopman et al. 2006; Camera et al. 2010; Farnfield et al. 2011). While the exact mechanism of exercise-induced mTORC1 activation has yet to be fully elucidated, muscle contraction has been reported to inactivate TSC2 through an Akt-independent activation of mTORC1 (Hornberger et al. 2004; Jacobs et al. 2013). mTORC1 activation subsequently phosphorylates mTOR and further downstream targets, p70S6k (ribosomal S6 kinase 1) and RPS6 (ribosomal protein S6) (Goodman 2014).

It remains unclear whether HV and HI resistance exercise protocols stimulate anabolic signaling in a similar or distinct manner. Multiple set resistance exercise elicits greater intramuscular anabolic signaling than single set exercise, indicating that exercise volume influences the muscle protein signaling response to exercise (Burd et al. 2010; Terzis et al. 2010). Low- versus high-intensity unilateral leg extensions performed to volitional fatigue have yielded inconsistent findings (Burd et al. 2010; Mitchell et al. 2012). Greater mTORC1 activation has also been demonstrated following a high volume (5 × 10 RM) versus a very low volume (15 × 1 RM) bilateral leg press protocol (Hulmi et al. 2012). Evidence appears to indicate that additional factors including muscle fiber recruitment (Gehlert et al. 2014), time-under-tension (Burd et al. 2012a), and metabolic stress (Popov et al. 2015) also influence intramuscular anabolic signaling. The optimal stimulus for maximizing the anabolic response from resistance exercise remains unclear. Thus, the purpose of this study was to examine intramuscular anabolic signaling in conjunction with circulating hormone concentrations following a typical HV and HI lower-body resistance exercise protocol in well-trained men. In addition, electromyography (EMG) analysis of the vastus lateralis was performed to examine muscle activation patterns between each resistance exercise protocol.

## Materials and Methods

### Participants

Ten resistance-trained men (24.7 ± 3.4 years; 90.1 ± 11.3 kg; 176.0 ± 4.9 cm; 14.1 ± 6.1% body fat) were recruited to participate in this randomized, cross-over design research study. Inclusion criteria required participants to be between the ages of 18 and 35 years, a minimum of 1 year of resistance training experience, and the ability to squat a weight equivalent to their body mass. Participants had 6.7 ± 4.6 years of resistance training experience with an average maximum barbell back squat of 172.7 ± 25.2 kg. All participants were familiar with the exercises and training intensities employed in the study. All participants were free of any physical limitations that may affect performance. In addition, all participants were free of any medications and performance-enhancing drugs, as determined by a health and activity questionnaire. Following an explanation of all procedures, risks, and benefits, each participant provided his informed consent prior to participation in this study. The research protocol was approved by the New England Institutional Review Board prior to participant enrollment.

### Maximal strength testing

Prior to experimental trials, participants reported to the Human Performance Laboratory (HPL) to establish maximal strength (1-RM) on all lifts involved in the exercise protocol. Prior to maximal strength testing, participants performed a standardized warm-up consisting of 5 min on a cycle ergometer against a light resistance, 10 body weight squats, 10 body weight walking lunges, 10 dynamic walking hamstring stretches, and 10 dynamic walking quadriceps stretches. The 1-RM test for the barbell back squat and leg press were performed using methods previously described (Hoffman 2006). Briefly, each participant performed two warm-up sets using a resistance of approximately 40–60% and 60–80% of his perceived maximum, respectively. For each exercise, 3–4 subsequent attempts were performed to determine the 1-RM. A 3–5 min rest period was provided between each attempt. For all other exercises, the 1-RM was assessed using a prediction formula based on the number of repetitions performed to fatigue using a given weight (Brzycki 1993). Attempts not meeting the range of motion criteria for each exercise or where proper technique was not used were discarded.

### Experimental trials

On the morning of each trial, participants reported to the HPL after a 10-h overnight fast and having refrained from all forms of moderate to vigorous exercise for the previous 72 h. Experimental trials were performed in a balanced, randomized order, and each experimental trial was separated by a minimum of 1 week to ensure adequate recovery. Each participant performed experimental trials at the same time of day to avoid diurnal variations. Participants provided a urine sample upon arrival to the HPL for analysis of urine specific gravity (USG) by refractometry to ensure an adequate hydration status (USG ≤ 1.020 defined as euhydration).

During each experimental trial, participants performed the standardized warm-up routine described above, followed by a lower-body resistance exercise protocol. Table1 depicts the HV and HI resistance exercise protocols. The HV protocol utilized a load of 70% 1-RM for sets of 10–12 repetitions with a 1-min rest period length between sets and exercises. The HI protocol utilized a load of 90% 1-RM for sets of 3–5 repetitions with a 3-min rest period length between sets and exercises. Both protocols included six sets of barbell back squats and four sets of bilateral leg press, bilateral hamstring curls, bilateral leg extensions, and seated calf raises. During each protocol, participants were verbally encouraged to complete each set. If the participant was unable to complete the desired number of repetitions, spotters provided assistance until the participant completed the remaining repetitions. Subsequently, the load for the next set was adjusted so that participants were able to perform the specific number of repetitions for each set.

**Table 1:** Resistance Exercise Protocols. The HV protocol utilized a load of 70% of one repetition maximum (1-RM) for sets of 10–12 repetitions with a 1 min rest period length between sets and exercises. The HI protocol utilized a load of 90% 1-RM for sets of 3–5 repetitions with a 3 min rest period length between sets and exercises. If the participant was unable to complete the desired number of repetitions, spotters provided assistance until the participant completed the remaining repetitions. Subsequently, the load was adjusted so that participants were able to perform the specific repetitions for each set

Exercise Order	High Volume Protocol (HV)			High Intensity Protocol (HI)		
	Sets × Repetitions	Intensity	Rest Time	Sets × Repetitions	Intensity	Rest Time
1. Barbell Back Squats	6 × 10–12	70% 1-RM	1 min	6 × 3–5	90% 1-RM	3 min
2. Bilateral Leg Press	4 × 10–12			4 × 3–5		
3. Bilateral Hamstring Curls	4 × 10–12			4 × 3–5		
4. Bilateral Leg Extensions	4 × 10–12			4 × 3–5		
5. Seated Calf Raises	4 × 10–12			4 × 3–5		

Following each resistance exercise protocol, participants remained in the laboratory for all postexercise assessments. Blood samples were obtained at six time points over the course of the study: baseline (BL), immediately postexercise (IP), 30 min postexercise (30P), 1 h postexercise (1H), 2 h postexercise (2H), and 5 h postexercise (5H). Fine needle muscle biopsies were completed at BL, 1H, and 5H.

To control for diet, participants were provided a standardized low protein, low carbohydrate breakfast bar (Atkins Nutritionals, Inc., Denver, CO: 7 g protein, 3 g carbohydrate, and 3 g fat) following BL assessments. Immediately following IP blood sampling, participants were also provided a flavored drink (355 mL, 0 g protein, 2.5 g carbohydrates, 0 g fat) to limit any nutritional impact on the signaling response. Participants were permitted to drink water ad libitum during experimental trials, and volume of water consumption was recorded.

### Muscle activation

To investigate muscle activation, EMG analysis of the vastus lateralis of the nondominant leg was assessed during every repetition for the multijoint exercises (barbell back squat and bilateral leg press) during each resistance exercise protocol. A bipolar surface electrode arrangement (Quinton, Milwaukee, WI) was placed at two-thirds of the line between the anterior superior iliac spine and superior lateral aspect of the patella, with the reference electrode placed over the tibial tuberosity. The skin beneath the electrodes was shaved, abraded, and cleaned with alcohol to keep interelectrode impedance below 5000 ohms. EMG signals were obtained with a differential amplifier (MP150 BIOPAC Systems, Inc., Santa Barbara, CA) sampled at 1000 Hz. Data were sent in real time to a computer via bluetooth and recorded for later analysis. To eliminate variance, all EMG preparation and electrode attachment was conducted by a single technician, and the foot placement and anatomical positioning of participants were recorded and kept consistent during each experimental trial. EMG signals were band-pass filtered from 10 Hz to 500 Hz and expressed as root mean square amplitude values by software (AcqKnowledge v4.2, BIOPAC Systems, Inc.). The average root mean square (RMS; microvolts) was calculated for each repetition by the software. For normalizing EMG analysis, maximal voluntary isometric contraction (MVIC) of the bilateral leg extension was obtained during the maximal strength testing visit (Burden 2010). All RMS values were normalized as a percent of MVIC. Test–retest reliability for the RMS of MVIC of the bilateral leg extension in our laboratory has been established (ICC = 0.88). MVIC was conducted in a bilateral leg extension machine with the knees flexed at 105.6 ± 4.2° and hands grasping the handlebars for stability. Participants were asked to extend the knee exerting maximal force against an immoveable resistance for 5 sec. The highest MVIC EMG value was used as the reference with which to normalize EMG signals. EMG data were reported as percentage of MVIC.

### Blood measurements

During each experimental trial, blood samples were obtained using a Teflon cannula placed in a superficial forearm vein using a three-way stopcock with a male luer lock adapter and plastic syringe. The cannula was maintained patent using an isotonic saline solution (Becton Dickinson, Franklin Lakes, NJ). BL blood samples were obtained following a 15-min equilibration period. IP blood samples were taken within 1 min of exercise cessation. Participants were instructed to lie in a supine position for 15 min prior to 30P, 1H, 2H, and 5H blood draws.

All blood samples were collected into three 6 mL Vacutainer® tubes. Blood samples were drawn into either plain, sodium heparin, or K_2_EDTA treated tubes. A small aliquot of whole blood was removed and used for determination of hematocrit and hemoglobin concentrations. The blood in the plain tube was allowed to clot at room temperature for 30 min and subsequently centrifuged at 3000 × *g* for 15 min along with the remaining whole blood from the other tubes. The resulting serum and plasma was placed into separate microcentrifuge tubes and frozen at −80°C for later analysis.

### Biochemical analysis

Blood lactate concentrations were analyzed from plasma using an automated analyzer (Analox GM7 enzymatic metabolite analyzer, Analox Instruments USA, Lunenburg, MA). Hematocrit concentrations were analyzed from whole blood via microcentrifugation (CritSpin, Westwood, MA) and microcapillary technique. Hemoglobin concentrations were analyzed from whole blood using an automated analyzer (HemoCue, Cypress, CA). Plasma volume shifts were calculated using the formula established by Dill and Costill (1974). To eliminate interassay variance, all samples were analyzed in duplicate by a single technician. Coefficient of variation for each assay was 1.4% for blood lactate; 0.4% for hematocrit; and 0.6% for hemoglobin.

Circulating concentrations of insulin-like growth factor-1 (IGF-1), insulin, testosterone, growth hormone (GH), and cortisol were assessed via enzyme-linked immunosorbent assays (ELISA) and a spectrophotometer (BioTek Eon, Winooski, VT) using commercially available kits. Myoglobin concentrations were determined via ELISA (Calbiotech, Spring Valley, CA) and a spectrophotometer. Lactate dehydrogenase (LDH) concentrations were determined via ELISA (Sigma-Aldrich, St. Louis, MO) and a spectrophotometer. To eliminate interassay variance, all samples for each assay were thawed once and analyzed in duplicate in the same assay run by a single technician. Coefficient of variation for each assay was 6.5% for IGF-1; 8.1% for insulin; 4.8% for testosterone; 4.9% for GH; 5.3% for cortisol; 4.1% for myoglobin; and 4.8% for LDH.

### Fine needle muscle biopsy procedure

Fine needle muscle biopsies were performed on the vastus lateralis muscle of the participant’s dominant leg using a spring-loaded, reusable instrument with 14-gauge disposable needles and a coaxial introducer (Argon Medical Devices Inc., Plano, TX). Following local anesthesia with 2 mL of 1% lidocaine applied into the subcutaneous tissue, a small incision to the skin was made and an insertion cannula was placed perpendicular to the muscle until the fascia was pierced. The biopsy needle was inserted through the cannula and a muscle sample was obtained by the activation of a trigger button, which unloaded the spring and activated the needle to collect a muscle sample. Multiple biopsy passes at each time point were made with the cannula in place, thus avoiding repeated skin punctures. Each muscle sample was removed from the biopsy needle using a sterile scalpel and was subsequently placed in a cryotube, rapidly frozen in liquid nitrogen, and stored at −80°C. A licensed physician performed all muscle biopsies.

### Intramuscular anabolic signaling analysis

Tissue samples were thawed and kept on ice for preparation and homogenization. A lysis buffer with protease inhibitor (EMD Millipore, Billerica, MA) was added to each sample at a rate of 500 *μ*L per 10 mg of tissue. Samples were homogenized using a Teflon pestle and sonication (Branson, Danbury, CT). Tissue samples were then placed on a plate shaker (Thermo Fisher Scientific Inc., Waltham, MA) for 10 min at 4°C and subsequently centrifuged at 10,000 × *g* for 5 min. The supernatant was aspirated and used for analysis.

Multiplex ELISA was used to quantitate the phosphorylation status of proteins specific to the mTORC1 signaling pathway using MAGPIX® (Luminex, Austin, TX) and a multiplex signaling assay kit (EMD Millipore) according to manufacturer’s guidelines. Multiplex ELISA has been validated (Hwang 2011) and previously used to determine the phosphorylation status of proteins in the mTORC1 signaling pathway (Sharma et al. 2012a,b; Gonzalez et al. 2015). Samples were analyzed for phosphorylation of IGF-1 receptor (IGF1R) at Tyr 1135/1136, insulin receptor (IR) at Tyr 1162/1163, insulin receptor substrate 1 (IRS1) at Ser 636, TSC2 at Ser 939, Akt at Ser 473, mTOR at Ser 2448, p70S6k at Thr 412, and RPS6 at Ser 235/236. The specificity of the p70S6k antibody recognized p70S6k I phosphorylated on Thr 412 and the splice variant p70S6k II phosphorylated on Thr 389. Total protein quantitation was conducted using a detergent compatible protein assay kit (Bio-Rad, Hercules, CA). Homogenized samples were diluted prior to being loaded and results are reported as fluorescence intensity expressed relative to total protein content. To eliminate interassay variance, all tissue samples were thawed once and analyzed in duplicate in the same assay run by a single technician. The average coefficient of variation for phospho-protein analysis was 8.4%.

### Dietary logs

Participants were instructed to maintain their normal dietary intake leading up to experiment trials. Participants were then instructed to record as accurately as possible everything they consumed during the 24 h prior to the first experimental trial. For the following experimental trial, participants were required to duplicate the content, quantity, and timing of their daily diet during the 24 h prior. Participants were instructed not to eat or drink (except water) within 10 h of reporting to the HPL for experimental trials.

### Statistical analysis

Prior to statistical procedures, all data were assessed for normal distribution, homogeneity of variance, and sphericity. If the assumption of sphericity was violated, a Greenhouse–Geisser correction was applied. Biochemical changes were analyzed using a two factor (trial × time) analysis of variance (ANOVA) with repeated measures on time. In the event of a significant F ratio, LSD post hoc tests were used for pairwise comparisons. Area under the curve (AUC) was also calculated for biochemical measures using a standard trapezoidal technique. AUC analysis was analyzed via paired samples *t*-tests. Mean muscle activation of each set of squat and leg press were analyzed using a two factor (trial × set) ANOVA. For effect size, partial eta squared statistics were calculated, and according to Green et al. (2000), 0.01, 0.06, and 0.14 were interpreted as small, medium, and large effect sizes, respectively. Significance was accepted at an alpha level of *P* ≤ 0.05 and all data are reported as mean ± SD.

## Results

### Resistance exercise protocol

All participants were adequately hydrated (USG ≤ 1.020) prior to each trial, and no significant differences were noted between trials for baseline USG (*P* = 0.98). No significant differences were noted for water consumption during each protocol (*P* = 0.34). As anticipated, significant differences between trials were noted for workout volume (*P* = 0.01). Workout volume (sets × load × reps) was significantly greater for HV (45300.0 ± 13919.8 kg) compared to HI (33633.5 ± 5661.9 kg).

### Electromyography analysis

**Figure 1:**
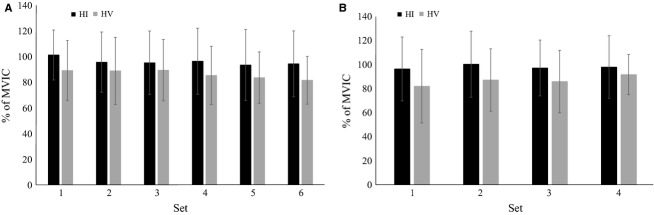
(A) Muscle activation during the squat exercise. (B) Muscle activation during the leg press exercise. HI, High intensity resistance exercise protocol; HV, High volume resistance exercise protocol; MVIC, Maximal voluntary isometric contraction. All data are reported as means ± SD.

Analysis of muscle activation during the squat exercise revealed no significant effect across the six sets (*F* = 3.0; *P* = 0.07; *η*^2^ = 0.16), and no significant interactions were noted (*F* = 1.1; *P* = 0.36; *η*^2^ = 0.07) (Fig.1A). In addition, no significant differences were noted in muscle activation during each of the four sets of leg press (*F* = 2.3; *P* = 0.09; *η*^2^ = 0.12), and no significant interactions were noted (*F* = 1.3; *P* = 0.27; *η*^2^ = 0.07) (Fig.1B).

### Biochemical analysis

**Figure 2:**
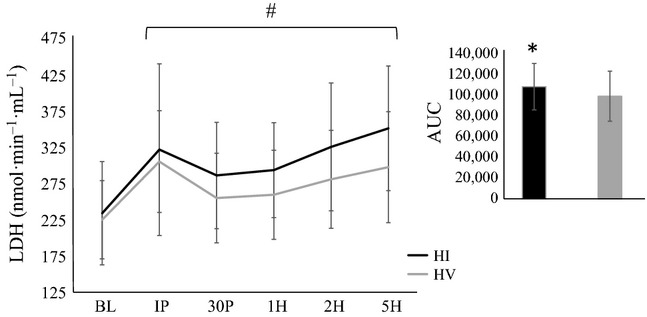
Lactate dehydrogenase (LDH) concentration following resistance exercise. Groups: HI, High intensity resistance exercise protocol; HV, High volume resistance exercise protocol. Time points: BL, Baseline; IP, Immediately post; 30P, 30-min post; 1H, 1-h post; 2H, 2-h post; 5H, 5-h post. Inset: area under the curve (AUC). All data are reported as means ± SD. *Significant difference between HI and HV (P ≤ 0.05). ^#^Significant difference from BL (P ≤ 0.05).

**Figure 3:**
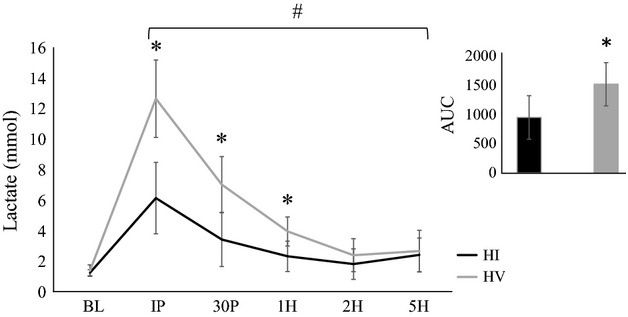
Lactate concentration following resistance exercise. Groups: HI=High intensity resistance exercise protocol; HV, High volume resistance exercise protocol. Time points: BL, Baseline; IP, Immediately post; 30P, 30-min post; 1H, 1-h post; 2H, 2-h post; 5H, 5-h post. Inset: area under the curve (AUC). All data are reported as means ± SD. *Significant difference between HI and HV (P ≤ 0.05). ^#^Significant difference from BL (P ≤ 0.05)

Significant time effects were noted for myoglobin (*F* = 46.7; *P* = 0.0001; *η*^2^ = 0.72), LDH (*F* = 13.1; *P* = 0.0001; *η*^2^ = 0.42), and lactate (*F* = 154.3; *P* = 0.0001; *η*^2^ = 0.90). Myoglobin concentrations (Table2) were significantly elevated from BL at IP, 30P, and 1H (*P* < 0.001). LDH and lactate concentrations (Figs.2, 3, respectively) were significantly elevated from BL at all time points (*P* < 0.001). Significant interactions were also noted for myoglobin (*F* = 5.8; *P* = 0.02; *η*^2^ = 0.25) and lactate (*F* = 27.5; *P* = 0.0001; *η*^2^ = 0.60), however, no significant interactions were noted for LDH (*F* = 0.8; *P* = 0.53; *η*^2^ = 0.04). Myoglobin concentrations were significantly greater during HI compared to HV at both IP (*P* = 0.02) and 30P (*P* = 0.01), whereas lactate concentrations were significantly greater during HV compared to HI at IP (*P* = 0.0001), 30P (*P* = 0.0001), and 1H (*P* = 0.001). AUC analysis indicated myoglobin (BL-1H) and LDH concentrations during HI were significantly greater than HV (*P* = 0.02 and *P* = 0.01, respectively). In addition, AUC analysis indicated that lactate concentrations during HV were significantly greater than HI (*P* = 0.003).

**Table 2:** Myoglobin concentration following resistance exercise. Groups: HI, High intensity resistance exercise protocol; HV, High volume resistance exercise protocol. Time points: BL, Baseline; IP, Immediately post; 30P, 30-min post; 1H, 1-h post

	BL	IP*†	30P*†	1H†
HI	29.3 ± 8.6	164.3 ± 93.5	201.6 ± 106.8	199.9 ± 104.3
HV	35.0 ± 13.4	91.9 ± 26.1	104.9 ± 34.5	141.9 ± 51.0

All data are reported as means ± SD.

* Significant difference between HI and HV (*P* *≤* 0.05).

† Significant difference from BL (*P* ≤ 0.05).

### Hormonal analysis

**Figure 4:**
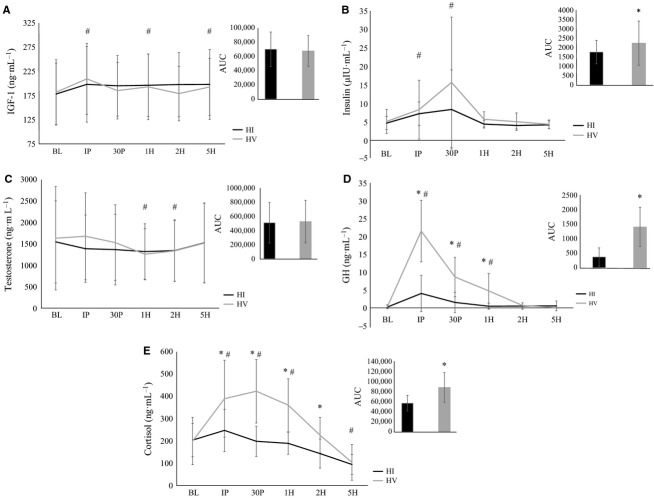
(A) Insulin-like growth factor-1 (IGF-1) (B) insulin (C) testosterone (D) growth hormone (GH) and (E) cortisol concentration following resistance exercise. Groups: HI, High intensity resistance exercise protocol; HV, High volume resistance exercise protocol. Time points: BL, Baseline; IP, Immediately post; 30P, 30-min post; 1H, 1-h post; 2H, 2-h post; 5H, 5-h post. Inset: area under the curve (AUC). All data are reported as means ± SD. *Significant difference between HI and HV (P ≤ 0.05). ^#^Significant difference from BL (P ≤ 0.05).

Hormonal concentrations following resistance exercise are depicted in Figure4. Significant time effects were observed for IGF-1 (*F* = 5.2; *P* = 0.0001; *η*^2^ = 0.23), insulin (*F* = 5.6; *P* = 0.03; *η*^2^ = 0.24), testosterone (*F* = 5.1; *P* = 0.02; *η*^2^ = 0.22), GH (*F* = 44.5; *P* = 0.0001; *η*^2^ = 0.71), and cortisol (*F* = 29.2; *P* = 0.0001; *η*^2^ = 0.62). IGF-1 concentrations were significantly elevated from BL at IP (*P* = 0.0001), 1H (*P* = 0.02), and 5H (*P* = 0.02). Insulin concentrations were significantly elevated from BL at IP (*P* = 0.004) and 30P (*P* = 0.02). Testosterone concentrations were significantly decreased from BL at 1H (*P* = 0.04) and 2H (*P* = 0.03), while GH and cortisol concentrations were significantly elevated from BL at IP (*P* = 0.0001 and *P* = 0.001, respectively), 30P (*P* = 0.0001 and *P* = 0.001, respectively), and 1H (*P* = 0.01 and *P* = 0.01, respectively). In addition, cortisol concentrations were significantly decreased from BL at 5H (*P* = 0.0001). Significant interactions were noted for GH (*F* = 22.4; *P* = 0.0001; *η*^2^ = 0.56) and cortisol (*F* = 8.4; *P* = 0.0001; *η*^2^ = 0.32), however, no significant interactions were noted for IGF-1 (*F* = 2.3; *P* = 0.06; *η*^2^ = 0.11), insulin (*F* = 1.1; *P* = 0.31; *η*^2^ = 0.06), or testosterone (*F* = 1.6; *P* = 0.22; *η*^2^ = 0.08). GH and cortisol concentrations were significantly greater during HV compared to HI at IP (*P* = 0.0001 and *P* = 0.01, respectively), 30P (*P* = 0.0001 and *P* = 0.001, respectively), and 1H (*P* = 0.02 and *P* = 0.003, respectively), while cortisol concentrations were also significantly greater during HV compared to HI at 2H (*P* = 0.02). AUC analysis indicated that the insulin, GH, and cortisol response during HV was significantly greater than HI (*P* = 0.04, *P* = 0.0001, and *P* = 0.003, respectively), however, no significant differences between trials were noted for IGF-1 or testosterone AUC (*P* = 0.39 and *P* = 0.44, respectively).

Relative to BL, plasma volume shifts were significantly different between trials at IP (*P* = 0.02). The difference between trials was not significant for any other time point. During HV, plasma volume decreased at IP, −8.0 ± 7.7%; increased at 30P, 2.1 ± 9.4%; increased at 1H, 7.2 ± 14.0%; increased at 2H, 3.7 ± 5.0%; and decreased at 5H, −1.6 ± 5.5%. During HI, plasma volume decreased at IP, −1.6 ± 3.1%; increased at 30P, 3.3 ± 3.6%; increased at 1H, 4.0 ± 3.0%; increased at 2H, 7.2 ± 7.3%; and decreased at 5H, −2.6 ± 4.0%. Blood variables were not corrected for plasma volume shifts due to the importance of molar exposure at the tissue receptor level.

### Intramuscular anabolic signaling

**Figure 5:**
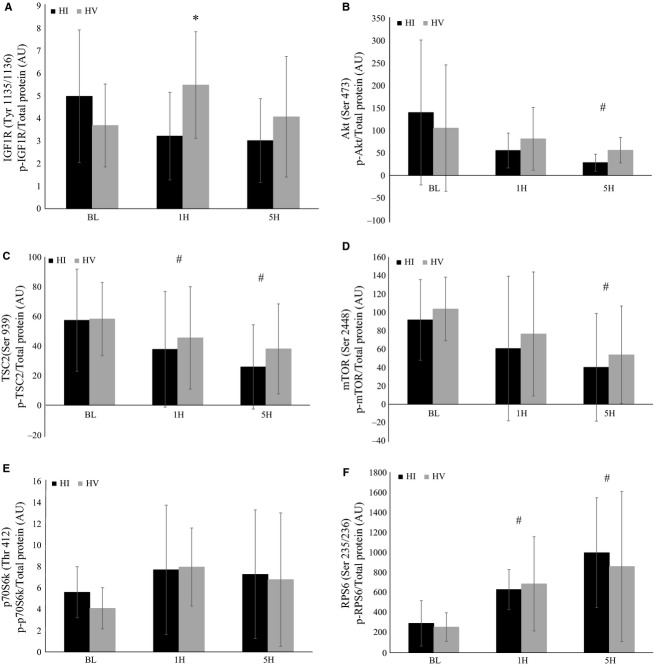
Phosphorylation of (A) IGF1R (Tyr 1135/1136) (B) Akt (Ser 473) (C) TSC2 (Ser 939) (D) mTOR (Ser 2448) (E) p70S6k (Thr 412) (F) RPS6 (Ser 235/236). Groups: HI, High intensity resistance exercise protocol; HV, High volume resistance exercise protocol. Time points: BL, Baseline; IP, Immediately post; 30P, 30-min post; 1H, 1-h post; 2H, 2-h post; 5H, 5-h post. Phosphorylation status of signaling proteins were determined relative to total protein concentration and are therefore reported as arbitrary units (AU). All data are reported as means ± SD. *Significant difference between HI and HV (P ≤ 0.05). ^#^Significant difference from BL (P ≤ 0.05).

Intramuscular anabolic signaling following resistance exercise is depicted in Figure5. No significant differences over time were noted for phosphorylation of IGF1R (*F* = 1.1; *P* = 0.35; *η*^2^ = 0.06), IR (*F* = 0.1; *P* = 0.95; *η*^2^ = 0.003), IRS1 (*F* = 1.3; *P* = 0.29; *η*^2^ = 0.07), or p70S6k (*F* = 2.4; *P* = 0.11; *η*^2^ = 0.12), and no significant interactions were noted for phosphorylation of IR (*F* = 1.4; *P* = 0.26; *η*^2^ = 0.07), IRS1 (*F* = 0.1; *P* = 0.88; *η*^2^ = 0.01), or p70S6k (*F* = 0.2; *P* = 0.82; *η*^2^ = 0.01). However, significant interactions were noted for phosphorylation of IGF1R (*F* = 4.1; *P* = 0.02; *η*^2^ = 0.19). Phosphorylation of IGF1R was significantly greater during HV compared to HI at 1H (*P* = 0.03).

Significant time effects were observed for phosphorylation of Akt (*F* = 4.4; *P* = 0.04; *η*^2^ = 0.20), TSC2 (*F* = 5.4; *P* = 0.01; *η*^2^ = 0.23), mTOR (*F* = 4.5 *P* = 0.02; *η*^2^ = 0.21), and RPS6 (*F* = 12.3; *P* = 0.0001; *η*^2^ = 0.41). Phosphorylation of TSC2 was significantly decreased from BL at 1H (*P* = 0.04), while phosphorylation of TSC2, Akt, and mTOR were significantly decreased from BL at 5H (*P* = 0.01, *P* = 0.02, and *P* = 0.01, respectively). Phosphorylation of RPS6 was significantly elevated from BL at 1H (*P* = 0.001) and 5H (*P* = 0.0001). However, no significant interactions were noted for phosphorylation of Akt (*F* = 0.8; *P* = 0.40; *η*^2^ = 0.04), TSC2 (*F* = 0.3; *P* = 0.78; *η*^2^ = 0.01), mTOR (*F* = 0.002; *P* = 0.99; *η*^2^ = 0.0001), or RPS6 (*F* = 0.3; *P* = 0.77; *η*^2^ = 0.02).

## Discussion

Resistance exercise initiates a multifaceted biochemical response regulating muscle protein synthesis and growth. In this study, signaling proteins within the mTORC1 pathway were examined in conjunction with circulating hormonal concentrations following two different lower-body resistance exercise protocols in resistance-trained men. The HV and HI protocol design was typical of specific mesocycles (e.g., hypertrophy and strength, respectively) used by strength/power athletes in their periodized training program (ACSM, 2009; Hoffman et al. 2009). Although workout volume was designed to be different a priori, both protocols required participants to use an intensity load that required maximal effort to achieve the required repetition range (i.e., reach momentary muscular failure). This effort appeared to cause greater changes in markers of muscle damage (i.e., myoglobin and LDH concentrations) during HI, but greater changes in a metabolic marker of stress (i.e., lactate concentration) during HV. Significant differences in the endocrine response were also observed between protocols. GH, cortisol, and insulin responses were significantly greater during HV than HI, however, no differences between protocols were observed for either the IGF-1 or testosterone response. Intramuscular anabolic signaling analysis revealed that only the phosphorylation of IGF1R at 1H was significantly greater during HV than HI, while no other differences were noted in the phosphorylation of all other signaling proteins between HV and HI.

The intensity used during each resistance exercise protocol produced similar muscle activation across sets in both the squat and leg press exercises. Muscle activation is influenced by the firing rate and number of motor units activated (Fuglsang-Frederiksen and Rønager 1988), and motor units appear to be recruited in accordance with the size principle during voluntary muscle contraction (Henneman et al. 1965). However, it has been suggested that lighter loads (20–30% 1-RM) lifted to momentary muscular failure will result in a similar amount of muscle fiber recruitment compared with heavier loads (50–80% 1-RM), thus promoting similar muscular adaptations (Burd et al. 2012b; Mitchell et al. 2012; Barcelos et al. 2015). In addition, the relationship between intensity and muscle protein synthesis may reach a plateau between intensities of ∼60–90% of 1-RM (Kumar et al. 2009). The results of this study indicated that HV and HI elicited similar muscle activation; however, it is important to note that during HV, considering the greater volume of training, the muscle activation was provided for a longer period of time.

Resistance exercise can induce significant microtrauma to muscle fibers (Nosaka et al. 2003). Myoglobin and LDH concentrations have been used extensively as markers of muscle damage and may indicate the integrity of the muscle cell membrane (Nosaka et al. 2003; Jamurtas et al. 2005; Rodrigues et al. 2010; Gonzalez et al. 2014). Although both HV and HI protocols elicited significant elevations in these markers, greater changes in myoglobin and LDH concentrations were observed following HI. While microtrauma to skeletal muscle fibers is accompanied by an inflammatory response, indirect markers of muscle damage have not shown to be a consistent indicator of exercise-mediated adaptation (Brentano and Martins 2011). Furthermore, muscle hypertrophy has been observed in the relative absence of muscle damage (Brentano and Martins 2011; Flann et al. 2011). Although both protocols elicited significant increases in circulating myoglobin and LDH concentrations, the role of exercise-induced elevations of markers of muscle damage in promoting gene expression influencing skeletal muscle adaptation remains unclear. Despite differences in markers of muscle damage between trials, intramuscular anabolic signaling did not appear to differ between the protocols.

Exercise-induced metabolic stress may also play a role in acute activation of mTORC1 signaling. Metabolic stress results from exercise that primarily relies on anaerobic glycolysis as its major energy provider. Lactate directly affects muscle cells in vitro by increasing mTOR and p70S6k phosphorylation (Oishi et al. 2015), and elevations in blood lactate have previously been demonstrated to be weakly associated (*r* = 0.38) with intramuscular anabolic signaling following resistance exercise in trained men (Popov et al. 2015). Resistance exercise protocols that utilize moderate to high intensities (60-85% 1-RM) and volumes (3–6 sets), with relatively short rest intervals (<90 sec) appear to elicit the greatest increase in blood lactate concentrations (Kraemer et al. 1990, 1991; Gotshalk et al. 1997; Smilios et al. 2003; Linnamo et al. 2005; McCaulley et al. 2009; Rahimi et al. 2010). Furthermore, the lactate response following high volume resistance exercise programs have previously been shown to be significantly greater than high intensity resistance exercise programs (Smilios et al. 2003; McCaulley et al. 2009). In this study, elevated blood lactate concentrations were observed following HV and HI, however, the lactate response was greater following HV. Despite large differences in blood lactate concentrations between protocols, intramuscular anabolic signaling did not appear to be different. Lactate production may contribute to mTORC1 activation, however, the mechanisms by which metabolic stress influences anabolic signaling are not fully elucidated and warrant further investigation.

Acute program variables, including exercise intensity, volume, and rest, have been shown to influence the endocrine response following resistance exercise (Kraemer and Ratamess 2005). Regardless of training status or age, heavy resistance exercise appears to be a potent stimulus for acute increases in circulating anabolic hormones (Hakkinen and Pakarinen 1993; Kraemer et al. 1995, 1999; Goto et al. 2003; Ahtiainen et al. 2005; Linnamo et al. 2005; Beaven et al. 2008; Boroujerdi and Rahimi 2008; Villanueva et al. 2012). Furthermore, high volume, short rest resistance exercise protocols are associated with greater elevations of GH (Kraemer et al. 1990; Smilios et al. 2003), testosterone (Crewther et al. 2008; McCaulley et al. 2009), and cortisol (Smilios et al. 2003; Crewther et al. 2008; McCaulley et al. 2009; Uchida et al. 2009) when compared to high intensity, long rest resistance exercise protocols. The results of this present study appear to be consistent with some, but not all of the previous investigations. The GH, cortisol, and insulin response to exercise was significantly greater following HV compared to HI, while no significant differences between the protocols were observed for IGF-1 or testosterone. Nevertheless, the role of transient hormonal increases in the adaptive response to resistance exercise is not well understood (Schroeder et al. 2013). It has been suggested that elevations in circulating concentrations of these hormones increase the likelihood of hormone-receptor interaction and thus enhance the probability of a physiological effect (Kraemer et al. 1990; Ahtiainen et al. 2003; Kraemer and Ratamess 2005). However, the mechanisms of exercise-mediated muscle hypertrophy have been suggested to be solely an intrinsic process, which may not be influenced by transient changes in circulating hormones (West et al. 2009, 2010a,b; Mitchell et al. 2013).

To the best of our knowledge, this appears to be the first study to compare intramuscular anabolic signaling responses following HV and HI resistance exercise paradigms that are typically used by strength/power athletes. mTORC1 signaling analysis revealed a greater phosphorylation of IGF1R at 1H following HV compared to HI, while the phosphorylation status of all other signaling proteins did not appear to be different between the two training protocols. However, the IGF1R may not be necessary for resistance exercise-induced mTORC1 signaling and muscle growth (Spangenburg et al. 2008). Using a transgenic mouse model, Spangenburg and colleagues (2008) reported that both Akt and p70S6k activation can be induced independent of a functioning IGF-1 receptor. Downstream signaling proteins, including mTOR, p70S6k, and RPS6, appeared to have similar activation patterns following HV and HI. Both resistance exercise protocols resulted in significant elevations in RPS6 phosphorylation, while not stimulating any change in p70S6k phosphorylation. The lack of any significant change in p70S6k phosphorylation following both resistance exercise protocols may be related to the greater training experience and muscle strength of the participants (Gonzalez et al. 2015). Several studies have suggested that a greater training status can attenuate resistance exercise-induced intramuscular anabolic signaling (Coffey et al. 2006; Tang et al. 2008; Ogasawara et al. 2013). The protein kinase mTOR serves as a critical protein which confers signaling to p70S6k and several other downstream signaling molecules that regulate protein synthesis and skeletal muscle mass (Hornberger 2011; Goodman 2014). The phosphorylation of p70S6k regulates several factors involved in translation initiation and protein synthesis (Goodman 2014), and the phosphorylated state of p70S6k has been shown to significantly correlate with myofibrillar protein synthesis rates (*r* = 0.31–0.34) (Kumar et al. 2009; West et al. 2010a) and exercise-induced hypertrophy (*r* = 0.53–0.89) (Baar and Esser 1999; Terzis et al. 2008; Mayhew et al. 2009; Mitchell et al. 2013). Although the exact role of RPS6 in the regulation of protein synthesis remains unclear, RPS6 is a downstream target of p70S6k with the potential to regulate protein synthesis and is commonly used as an indirect marker of mTORC1 activation (Goodman 2014). Based upon the results of this study, it appears that HV and HI resistance exercise protocols elicit similar acute mTORC1 activation in resistance-trained men.

Despite significant differences in the endocrine response following HV and HI, both protocols stimulated similar mTORC1 activation following resistance exercise. Although it is well appreciated that hormones play an important role in regulating muscle mass, there is much discrepancy in the literature on the capacity of transient hormonal elevations to increase muscle protein synthesis in humans (Schroeder et al. 2013). Although the exact mechanism underlying increased mTORC1 activation following resistance exercise remains relatively elusive, mTORC1 has been suggested to be activated by increasing the activity of Rheb (Ras homolog enriched in brain) (Marcotte et al. 2015). mTORC1 activation requires phosphorylation of TSC2 (a negative regulator of Rheb), which subsequently causes TSC2 to be sequestered away from Rheb allowing mTORC1 to be activated (Marcotte et al. 2015). Resistance exercise and growth factors including insulin and IGF-1 lead to the phosphorylation of TSC2 (Inoki et al. 2002; Jacobs et al. 2013; Menon et al. 2014). When insulin and/or IGF-1 bind to their membrane receptors, TSC2 is subsequently phosphorylated via Akt (Inoki et al. 2002; Menon et al. 2014), whereas resistance exercise-induced activation of mTORC1 appears to be Akt-independent (Hornberger et al. 2004). It remains unclear what mediates TSC2 phosphorylation following resistance exercise (Marcotte et al. 2015). Nevertheless, resistance exercise and growth factors share the same final step in mTORC1 activation (via phosphorylation of TSC2) (Marcotte et al. 2015). Since the end result of both resistance exercise and growth factors is the movement of TSC2 away from Rheb via different upstream kinases, resistance exercise and hormonal exposure may not offer a synergistic effect. This appears to be consistent with the results of this study, in which the greater GH, cortisol, and insulin response following HV did not appear to augment intramuscular anabolic signaling. Furthermore, previous research has demonstrated that physiological fluctuations in anabolic hormones do not necessarily enhance muscle protein synthesis (West et al. 2009), intramuscular anabolic signaling (Spiering et al. 2008; West et al. 2009), or resistance training-induced muscle hypertrophy (Mitchell et al. 2013). The prominent role of acute increases in hormones such as GH and cortisol may be to meet a greater metabolic demand caused by the resistance exercise protocol, rather than promoting muscle protein synthesis.

This study investigated the acute anabolic response following two typical lower-body resistance exercise paradigms in experienced, resistance-trained men. The results of this study may reflect the lower adaptive ability among highly trained individuals, accounting for the attenuated signaling responses in comparison to untrained individuals (Coffey et al. 2006; Tang et al. 2008; Nader et al. 2014; Gonzalez et al. 2015). Although the stimulation of muscle protein synthesis appears to requires mTORC1 activation (Anthony et al. 2000; Kubica et al. 2005; Gundermann et al. 2014), a dissociation between anabolic signaling and muscle protein synthesis may exist (Greenhaff et al. 2008; Mitchell et al. 2015). Furthermore, acute measures of muscle protein synthesis may not fully explain the dynamic process of muscle hypertrophy consequent to resistance training (Damas et al. 2015). A potential limitation of this study is that time-under-tension, total work, and contraction velocity were not quantified during each protocol. In addition, the regulation of additional receptors (i.e., androgen receptor) was not examined following each protocol. We also recognize that the methods of studying intramuscular signaling in vivo in humans are accompanied by inherent limitations as it requires repeated biopsy sampling of a small population of muscle fibers at a few, distinctive time points following exercise and the analyzed tissue is assumed to be representative of the entire muscle.

In conclusion, HI appeared to cause greater changes in markers of muscle damage (e.g., myoglobin and LDH concentrations), but greater changes in lactate concentration were observed following HV. The GH, cortisol, and insulin response to exercise was significantly greater following HV than HI. However, the phosphorylation status of signaling proteins within mTORC1 was not significantly different between HV and HI, with the exception of IGF1R. Phosphorylation of IGF1R was significantly greater following HV at 1H compared to HI. Despite significant differences in lactate, myoglobin, LDH, and hormone concentrations following HV and HI, the regulation of signaling proteins within mTORC1 appeared to be similar following both protocols in resistance-trained men.
